# Editorial: Frontiers in Fungal Virus Research

**DOI:** 10.3389/fcimb.2019.00456

**Published:** 2020-01-22

**Authors:** Liying Sun, Nobuhiro Suzuki, Daohong Jiang, Massimo Turina, Jiatao Xie

**Affiliations:** ^1^State Key Laboratory of Crop Stress Biology for Arid Areas, College of Plant Protection, Northwest Agriculture and Forestry University, Yangling, China; ^2^Institute of Plant Science and Resources, Okayama University, Okayama, Japan; ^3^The State Key Laboratory of Agricultural Microbiology, Huazhong Agricultural University, Wuhan, China; ^4^Institute for Sustainable Plant Protection, Italian National Research Council, Rome, Italy

**Keywords:** mycovirus, identification, hypovirulence, transmission, host defense, virus-virus interaction

Compared to animal and plant viruses, fungal viruses have been relatively less studied. However, the recent development of associated methodologies including sequencing technologies has led to the rapid advancement of research on viruses infecting fungi or fungi-like organisms. Fungi can cause human/animal diseases, and they are the major causative agents of crop plant diseases. Fungi harbor highly diverse viruses, many of which constitute distinct virus families/groups that are not present in other organisms. Of note is that there are also some virus taxonomical groups comprising both plant and fungal viruses, suggesting an active and recent horizontal transfer of viruses between the two kingdoms. Therefore, fungal virology is particularly significant for its applicative, fundamental, and evolutionary implications. The Research Topic “Frontiers in Fungal Virus Research” is aimed to present the most recent progresses on different aspects of fungal virology. This topic contains 13 research and two review articles, covering a variety of topics including virus identification, virus-mediated modulation of fungal pathogenicity, virus-virus interactions, and host defense.

In this topic, nine research papers report the characterization of novel fungal viruses from various fungi. Khalifa and MacDiarmid identified a novel totivirus from *Trichoderma koningiopsis*. Another isolate belonging to the same species also was found from *Clonostachys rosea* distinct from *T. koningiopsis*, indicating a very recent natural horizontal transmission of this virus between unrelated fungi. It is interesting to further investigate how this virus is transmitted across different fungal species. Using RNA-seq analysis, Picarelli et al. revealed a high diversity of fungal viruses infecting *Rhizoctonia solani* isolated from *Zoysia japonica* (a cultivated grass) in Brazil. Most of the viruses show low sequence identity with other viruses, which include four large contigs of putative viral RNA that could not be assigned to any existing clade of viruses present in the databases. This suggests that they all belong to possible new viral species. This study provides a sound foundation for further understanding the diversity of fungal viruses. Kartali et al. identified novel totiviruses from *Umbelopsis ramanniana*. This work is particularly interesting and important because it is the first report on fungal viruses infecting this fungal genus, and this fungal species is an oleaginous fungus, a potential oil source for biodiesel production. Wang et al. characterized two novel viruses from *Sclerotinia sclerotiorum* each related to members of the order *Tymovirales* and the genus *Botybirnavirus*. Neither of them confers hypovirulence to the host. Moreover, they detected the accumulation of viral-derived siRNAs in *S. sclerotiorum*, indicating that RNA silencing operates against these viruses in the fungus. Li et al. characterized a novel hypovirulence-inducing hypovirus from *Alternaria alternata* isolated from an apple tree. Moreover, this virus also confers hypovirulence in *Botryosphaeria dothidea*, the causal fungal pathogen of apple white rot. Thus, this virus is a potentially valuable material as a bivalent biocontrol agent against fungal diseases in apple tree. Wang et al. identified a novel virus related to members of the genus *Chrysovirus*, family *Chrysoviridae* from *Penicillium crustosum*, a pathogen of citrus tree. Interestingly, this virus does not affect the growth or morphology of the fungal host but it decreases the fungal resistance against a fungicide. Certainly, this virus is a good molecular tool to study the mechanism of fungal resistance against fungicide. Jiang et al. identified a novel partitivirus from an opportunistic human pathogenic fungus, *Aspergillus flavus*. Infection by this virus causes significantly abnormal colonial and spore morphologies, but does not significantly affect its host virulence. It is important to further explore the presence of viruses in this important human-pathogenic fungus. Using RNA-seq analysis, Zhu et al. characterized viruses that are present in a hypovirulent strain of *Sclerotium rolfsii*. Interestingly, this fungal strain harbors various new viruses belonging to several established virus families and also some novel viruses that could not be assigned to any of the existing families or orders. Chun et al. identified a novel partitivirus from *Trichoderma harzianum*. *Trichoderma* species are known for their ability to inhibit other fungi, and therefore they are used as biocontrol agents for controlling common soil-borne plant pathogens. The important finding in this study is that although the infection by this partitivirus does not affect growth or morphology of its fungal host, it enhances antifungal activities of *T. harzianum* against other fungi, and this is associated with increased activity of antifungal enzyme, β-1,3-glucanase. It is necessary to further analyze how this partitivirus infection regulates the activity of a specific antifungal enzyme. Li et al. present a comprehensive review on fungal viruses infecting members of the fungal genus *Fusarium*. *Fusarium* species are important phytopathogenic fungi that cause damage in a wide variety of host plant species and also produce mycotoxin. Highly diverse viruses are widely present in *Fusarium* spp., and some of them induce hypovirulence in their fungal hosts. This review provides valuable knowledge for understanding virus diversity and prospects for development of biocontrol methods against *Fusarium* spp.

Two papers report studies on the roles of RNA silencing and autophagy pathways in fungal virulence and defense against virus infection in fungi. Neupane et al. revealed that the *Argonaute-like* (*agl*)-2 gene of *S. sclerotiorum* is important for virulence and host tolerance against virus infection. Intriguingly, deletion of *agl-2, agl-4, Dicer-like* (*dcl*)-*1*, and *dcl*-*2*, which are key components of RNA silencing, does not affect the production of the most abundant endogenous small RNAs in *S. sclerotiorum*. This suggests the existence of alternative enzymes/pathways for small RNA biogenesis in *S. sclerotiorum*. Autophagy is a highly conserved degradation mechanism of cellular constituents in eukaryotes. Shi et al. observed that infection by a hypovirus in *Cryphonectria parasitica* induces proliferation of autophagic-like vesicles. Furthermore, they demonstrated that *C. parasitica cpatg8*, a gene encoding a homolog of a key autophagy component, *Atg8*, of *Saccharomyces cerevisiae*, is important for fungal virulence and sporulation. Interestingly, *cpatg8* was also shown to play a positive role in hypovirus replication. Recently, autophagy is shown to have both anti- and pro-viral functions in other eukaryotic systems. This is a pioneering study that explores the involvement of autophagy in viral infection in filamentous fungi.

Using GFP-labeled strains of *Fusarium oxysporum*, Torres-Trenas et al. demonstrated that a hyphovirulence-inducing chrysovirus affects the speed and spatial distribution of fungal colonization into plant roots. This is the first study that clearly provides microscopic evidence that a mycovirus can influence the pattern of plant colonization by its fungal host. Kashif et al. examined the reciprocal effects of partitiviruses on the horizontal transmission efficiency through hyphal anastomosis in *Heterobasidion* spp. They observed that two viruses could synergistically enhance the overall transmission rate or antagonistically affect their transmission depending on specific virus combinations. Therefore, the interaction between co-infecting viruses and their host is complex, and the underlying mechanism remains to be elucidated. Thapa and Roossinck discussed determining factors for fungal-virus co-infection. Co-infections of natural fungal isolates by fungal viruses are commonly observed. The authors present interesting perspectives of how suppression of fungal non-self-recognition and RNA silencing, and nutritional/chemical compounds could affect the outcome of virus co-infection in fungi.

Overall, these works contain rich novel information from diverse viruses and fungal species regarding various topics of fungal virology. Indeed, they provide valuable research materials and interesting biological findings that warrant further detailed mechanistic studies. Mycovirus research will contribute to further advances of virology in general as well as lay a solid foundation for research development of control methods of many fungal diseases. Lastly, the editors of this topic greatly appreciate all of the authors and reviewers for their contributions to the collection.

## Author Contributions

LS, NS, DJ, MT, and JX edited the Research Topic of Frontier in Fungal virus Research and wrote the manuscript.

### Conflict of Interest

The authors declare that the research was conducted in the absence of any commercial or financial relationships that could be construed as a potential conflict of interest.

